# The impact of intermittent fasting during Ramadan on psychomotor and cognitive skills in adolescent athletes

**DOI:** 10.3389/fspor.2024.1362066

**Published:** 2024-06-05

**Authors:** Houda Bougrine, Nasr Chalghaf, Chiraz Azaiez, Ayat S Hammad, Ghada Boussayala, Moez Dhahri, Hamdi Henchiri, Ali Ibrahim Abd Ulwahid Al-Saedi, Mazin Dawood Ahmed Al-Hayali, Ahmed Wateed Mazyed Shdr AL-Rubaiawi, Ahmed Farooq Tawfeeq Ezzi, Nabee Muttlak Nasser AL-Sadoon, Nizar Souissi, Fairouz Azaiez, Ismail Dergaa, Maha Al-Asmakh

**Affiliations:** ^1^ High Institute of Sports and Physical Education of Gafsa, University of Gafsa, Gafsa, Tunisia; ^2^ Physical Activity Research Unit, Sport and Health (UR18JS01), National Observatory of Sports, Tunis, Tunisia; ^3^ Department of Health Sciences (DISSAL), Postgraduate School of Public Health, University of Genoa, Genoa, Italy; ^4^ Faculty of Human and Social Science of Sfax, University of Sfax, Sfax, Tunisia; ^5^ Higher Institute of Sport and Physical Education of Sfax, University of Sfax, Sfax, Tunisia; ^6^ Sociological Research Group on Contemporary Societies (GRESCO), University of Limoges, Limoges, France; ^7^ Department of Biomedical Science, College of Health Sciences, QU Health, Qatar University, Doha, Qatar; ^8^ Biomedical Research Center, Qatar University, Doha, Qatar; ^9^ Ministry of Education, General Directorate of Education in the Province of Maysan, Amarah, Iraq; ^10^ College of Physical Education and Sports Sciences, University of Karbala, Karbala, Iraq; ^11^ University of Baghdad, Baghdad, Iraq; ^12^ Aliraqia University, Baghdad, Iraq; ^13^ Training and Qualification Directorate, Ministry of Interior, Baghdad, Iraq; ^14^ Primary Health Care Corporation (PHCC), Doha, Qatar; ^15^ Higher Institute of Sport and Physical Education of Kef, University of Jendouba, El Kef, Tunisia

**Keywords:** cognitive functions, dietary intake, female athletes, ramadan intermittent fasting, Pittsburgh Sleep Quality Index, psychomotor performance, reaction time test, vigilance test

## Abstract

**Introduction:**

Intermittent fasting (IF) represents a dietary intervention similar to caloric restriction, characterized by the strategic limitation of food consumption. Among the diverse array of practices for IF, Ramadan IF (RIF), a religious observance in Islam, mandates that healthy adult Muslims abstain from both food and drinks during daylight hours. In sports, researchers have extensively studied IF effects on health, including sleep and physical performance, but its impact on cognitive functions during RIF remains understudied. Therefore, this study was conducted to evaluate the influence of RIF on psychomotor and cognitive performance among young female athletes.

**Methods:**

To achieve this purpose, a cohort of 23 female handball players, aged 17.2 ± 0.5 years, participated in a series of six testing sessions: one conducted prior to Ramadan (R0), and others during the first (R1), second (R2), third (R3), and fourth (R4) weeks of Ramadan, followed by a session in the week after Ramadan (R5). Each session involved assessments using a Simple Reaction Time Test (SRT), Choice Reaction Time Test (CRT), Vigilance Test (VT), and Mental Rotation Test (MRT). Additionally, dietary intake, body composition, and Pittsburgh Sleep Quality Index (PSQI) scores were evaluated during these periods.

**Results and discussion:**

The obtained data illustrated that there was a decrease in SRT, CRT, VT, and MRT performances during R1 in comparison to R0 (all *p* < .001). This reduction was also observed in R2, R3, R4, and R5. Notably, during the fourth week of Ramadan (R4), these cognitive and psychomotor parameters were significantly lower than during the earlier weeks (R1, R2, R3; all *p* < .001). Furthermore, a gradual decrease in total PSQI scores, sleep quality, and sleep duration was observed throughout the Ramadan period, reaching the lowest levels during R4. These findings illustrate that RIF has a significantly detrimental impact on neuromuscular and cognitive abilities as well as sleep quality in young female athletes. The study also highlights a fluctuating pattern in cognitive function across the four weeks of Ramadan, with the most pronounced decline observed during the final week of fasting illustrating the importance of conducting similar studies on normal individuals from both genders with larger sample size.

## 1 Introduction

Intermittent fasting (IF) has gained attention for its potential health benefits and impact on body composition, especially in patients with prevalent contemporary health issues (1). This diet, which is prevalent worldwide in various forms, includes specified periods for fasting (2). Examining various diets, such as IF, has grown in popularity among athletes as an approach that can improve their health, performance, and ability to adapt to exercise (3). Ramadan intermittent fasting (RIF), characterized by daily fasting from food and drink, presents a particular challenge that could affect athletic performance (2). The rising demands of modern sport, coupled with the increasing number of Muslim athletes in Western (non-Muslim) countries, has prompted sports scientists to study the effects of Ramadan intermittent fasting (RIF) on athletic performance during and after the month. In the holy month of 28 to 30 days, both pubertal healthy adults and adolescents refrained from consuming food, fluids, cigarettes, medications, and participating in sexual activity from dawn to sunset (4). Given the lunar basis of the Islamic calendar, Ramadan annually shifts, occurring in different times of the year, corresponding to various seasons and geographical locations (4). This temporal variability of Ramadan has led to its coincidence with major international sporting events. Notably, the 2014 FIFA World Cup [1435 Hijri Calendar (HC)] and the 2012 Olympics (1433 HC) both took place during Ramadan. Furthermore, the 2013 African Women's Junior Handball Championship (1434 HC), the 2016 Summer Olympic Games (1437 HC), the 2018 Mediterranean Games (1439 HC), and the 2018 FIFA World Cup (1439 HC) all took place immediately after this fasting month. Thus, the timing of those sports events presents unique challenges and consideration for the performance and well-being of Muslim athletes participating in these high-profile competitions. Crucially, this circumstance requires Muslim staff members to cope with this fasting period by maintaining the best productivity while fasting (5).

Recent publications indicate that the exclusive nighttime ingestion of enormous quantities of food during Ramadan, along with various lifestyle modifications, can adversely affect sleep quality and duration in athletic (6, 7) and non-athletic population, as well as impair physical performance (4, 8). The observed changes in dietary habits and sleep patterns during this period have prompted multiple researchers to investigate the impact of fasting on the athlete's physical performance. Interestingly, the majority of these studies have focused on the physical parameters, with less attention being given to the cognitive performance. While engaging in sports and exercise during RIF has been related to various benefits for physical, mental, and spiritual well-being, as well as community involvement, it has been also associated with various risks of dehydration, low blood sugar, fatigue, and delayed recovery (9). Furthermore, previous data did not support the association of RIF with any changes in dietary intake in both male (10) and female athletes (2, 11). However, an athlete's capacity to focus their cognitive resources on internal or external stimuli plays a pivotal role in achieving success particularly in team ball sports. In team ball sports, such as football and handball, optimizing performance encompasses a multifaceted approach that includes physical, technical, tactical, and cognitive abilities. Consequently, scientists become more interested in impact of RIF on mental health and cognitive function as factors in sports success particularly in team ball sports (12).

In this context, the effects of RIF on athletes' cognitive performance have been investigated in several studies, but the results were inconclusive, especially concerning young and/or female athletes. Some studies have reported no significant correlation between RIF and short-term memory performance (12, 13), motor reaction time (14), choice reaction time (15, 16), simple reaction time (16, 17), or attention (18, 19). Conversely, some investigations have indicated that RIF may be adversely associated impact processing speed (20), attention (4, 19), and simple reaction time (4, 21). To the best of our knowledge, only three studies (4, 12, 18) have examined the cognitive effects of RIF in female athletes. These include an investigation on young sprinters (12) and two studies on young handball players (4, 18). Although none of these studies explored cognitive performance separately from physical performance, the results remained inconclusive. While (4) reported a negative impact of RIF on cognitive performance, the other two studies did not observe any significant effects on alertness or vigilance. The variability in those findings may be attributed to individual factors such as sleep quality, training time and load, food, and the magnitude of lifestyle adjustments implemented by athletes and/or their coaches. Notably, the majority of conducted research in this field has concentrated on the male sex athletes, leaving a gap in understanding the influence of RIF on cognitive performance in female athletes. Thus, in our study, we aim to obtain a more comprehensive understanding for the impact of RIF on cognitive and psychomotor functions, particularly in young, and female athletes.

## 2 Materials and methods

### 2.1 Participants

The protocol of this study complied with Helsinki's declaration for human experimentation and was approved by the Ethics Committee of the Research Unit, Sportive Performance, and Physical Rehabilitation, High Institute of Sports and Physical Education, El Kef, University of Jendouba, Jendouba, Tunisia and the Higher Institute of Sport and Physical Education of Kef, El Kef (Tunisia) (CPP: 05/2022). It also complied with the ethical and procedural requirements of the journal for the conduct of sports medicine and exercise science research (22). All participants provided written informed consent prior to their participation, and in the case of minors, consent was also acquired from their parents or legal guardians. This process was conducted subsequent to a thorough briefing about the study's methodology, along with a discussion of the potential risks and benefits involved.

The estimated sample size for this study was calculated using G*Power software (23), following the guidelines outlined by (24). The alpha level was set at 0.05, and the desired statistical power at 0.80. Effect sizes were estimated to be 0.3, based on a similar study (4) and consensus among the authors. This analysis led to the conclusion that a minimum cohort of 20 athletes would be requisite to effectively mitigate the risk of incurring a Type II statistical error in the study.

Eligibility for participation in the study was confined to active, healthy female handball players, who have a minimum experience in sport of 3 years (5.3 ± 0.8 years) with an average 4.4 ± 0.5 training sessions per week. All participants were full-time athletes free from injuries or illnesses, non-smoke, and low consumers of caffeine. All athletes were categorized as low caffeine consumers (0.74 ± 0.1 mg·kg·day^−1^), with daily consumption less than 0.99 mg·kg·day^−1^, following a recent suggested classification (25) using a modified version of the Food Frequency Questionnaire (FFQ) (26) for the month preceding the commencement of the study (27). Additionally, they were required not to consume alcohol or drugs and to be free from any medication use. A prerequisite for participation was a history of observing Ramadan fasting for at least 3 years (4.3 ± 0.6 years).

Out of the 57 surveys examined, only 32 female team-sports players met the criteria and volunteered to participate in the study. Nevertheless, during the timepoints of the study, a total of nine participants dropped out. Among them, six athletes were excluded from the data collection due to their menstruation cycle (two in the first week, two in the second week, one in the third week, and one in the last week of RIF) during the timepoints of the study. This exclusion was conducted to ensure consistent fasting status among all participants to maintain methodological integrity and minimize variability, as in Islam they must refrain from fasting during menstruation. This exclusion was conducted to ensure consistent fasting status among all participants to maintain methodological integrity and minimize variability. Further, three athletes were excluded from the study: two due to injuries (one in the second week and one in the last week of RIF) and one for not completing all testing sessions resulting in a total number of 23 adolescent female handball players from the Tunisian League A. The average age, hight, weight, and body mass index (BMI) for our cohort were 17 ± 0.6 years, 1.7 ± 0.1 m, 62.3 ± 6.9 kg, and 21.7 ± 1.9 kg/m^2^, respectively. The detailed demographic and physical characteristics of the participants are presented in Table 1.

**Table 1:** General characteristics of the study subjects (*n* = 23).

	Minimum	Maximum	Mean
Age (years)	16	18	17 ± 0.6
Body mass (kg)	51	76	62.3 ± 6.9
Height (m)	1.6	1.8	1.7 ± 0.1
Body mass index (kg/m^2^)	18.1	26.3	21.7 ± 1.9
Fasting experience (years)	4	6	4.3 ± 0.6
MEQ questionnaire score	42	55	49 ± 4.4
Practice experience (years)	4	7	5.3 ± 0.8
Training sessions frequency/week	4	5	4.4 ± 0.5

MEQ, morningness-eveningness questionnaire of Horne and Ostberg (28). The minimum, maximum, mean, and standard deviation values of the participants’ characteristics are shown in table.

Regarding reproductive health, none of the participants reported menstrual or endocrine disorders during the last 3 months with a regular menstrual cycles length (28.2 ± 1.8 days) with a swing of no more than 3 days (29). They were also not using any contraceptive methods, including patches, oral contraceptives, injectables, implants, or intrauterine devices. To account for the phase of the menstrual cycle, which could influence the study's outcomes, the “My Calendar 3®” mobile application (Period Tracker) was used. This application is designed to identify and determine the different phases of the menstrual cycle, as outlined by (30). Additionally, the study considered the potential impact of circadian typology on the results. To this end, participants' chronotypes were assessed using the self-assessment questionnaires developed by (28) which assesses sleep and activity preferences using 19 items on a Likert scale. Players with extreme morning or evening tendencies were excluded. Only those who were classified as “neither type,” with scale scores ranging from 42 to 55 and without any sleep disorders, were included in the study. This criterion ensured a homogeneous group in terms of circadian preferences, which could otherwise have influenced the study's findings.

### 2.2 Experimental design

The research was carried out during the holy month of Ramadan, spanning from April 2nd to May 1st, 2022 (1443 HC). Throughout this period, daily fasting duration fluctuated between approximately 14.5 to 15.5 h, with dawn and sunset times ranging from 04:25–03:28 a.m. and 06:43–07:10 p.m. local time, respectively. In preparation for the study and to familiarizes the participants with the assessment protocols, they were invited to the indoor facility two weeks before the commencement of Ramadan.

**Figure 1:**
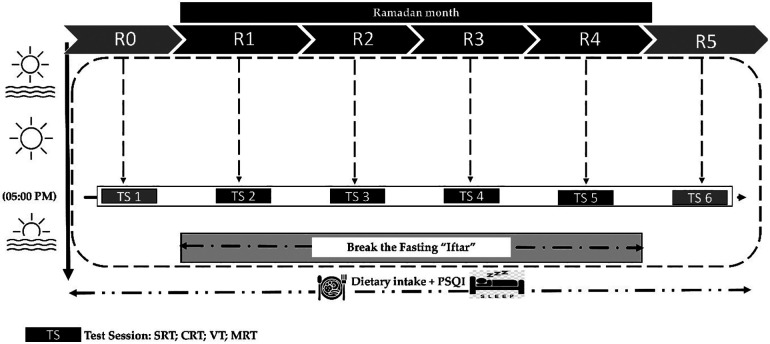
Study design: R0: during the week before Ramadan; R1: during the first week of Ramadan; R2: during the second week of Ramadan; R3: during the third week of Ramadan; R4: during the fourth week of Ramadan; R5: during the week following Ramadan. SRT, simple reaction time test; CRT, choice reaction time test; VT, vigilance test; MRT, mental rotation test; PSQI, Pittsburgh Sleep Quality Index questionnaire, all times given are expressed in local (GMT + 1 h).

The experimental sessions were scheduled across six distinct timepoints: the week before Ramadan (R0), the first week of Ramadan (R1), the second week of Ramadan (R2), the third week of Ramadan (R3), the fourth week of Ramadan (R4), and the week following Ramadan (R5). Each assessment was consistently conducted in the afternoon, between 05:00 p.m. and 06:00 p.m. During each session, participants sequentially performed four tests: The Simple Reaction Time Test (SRT), the Choice Reaction Time Test (CRT), the Vigilance Test (VT), and the Mental Rotation Test (MRT). A recovery period of 5 min was allowed between each test. Additionally, assessments of body mass, sleep quality, and dietary intake were conducted during each of these six periods (Figure 1).

Participants were instructed to maintain their regular physical activity routines throughout the study and to avoid any intense or strenuous exercises. All testing timepoints were held in the same indoor sports facility and at the same time of day (between 05:00 p.m. and 06:00 p.m.) under similar controlled conditions. The environmental conditions during timepoints were monitored, with average temperatures and humidity recorded as follows: 24°C (55% humidity) at R0, 26°C (46% humidity) at R1, 27°C (45% humidity) at R2, 28°C (46% humidity) at R3, 27°C (45% humidity) at R4, and 28°C (48% humidity) at R5.

#### 2.2.1 The Pittsburgh Sleep Quality Index (PSQI)

The subjective sleep quality over the six testing periods was evaluated using the validated Arabic version of the Pittsburgh Sleep Quality Index (31). This index consisted of 19 questions addressing seven aspects of sleep, including duration, quality, latency, efficiency, disturbances, daytime dysfunction, and the use of sleeping medications. The total score ranged from 0 to 21, with a score of “0” indicating no sleep-related issues and a score of “21” indicating severe issues across all areas of sleep. A global score lesser than 5 indicates good sleep quality. However, a global score of more or equal to 5 up to 21 indicates poor sleep quality (32).

#### 2.2.2 Dietary intake

The participants kept a food consumption diary, recording their dietary intake of food beverages and daily nutrient intake for each week in each of the six testing phases (R0, R1, R2, R3, R4, and R5). An experienced nutritionist conducted an interview with each participant to ensure the accuracy and completeness of the dietary records. The collected dietary data were then analyzed by the same nutritionist using the Bilnut program (Nutrisoft, Cerelles, France) along with the food composition tables of the National Institute of Statistics of Tunis (1978). We utilized a Tunisian manual of food photographs (FP 24-hR), depicting standard portion sizes of the foods commonly consumed in Tunisia to improve the estimation of consumed portions in a 24-h recall and recording the amount of food (33). This manual of food photography involves digital photographs of three different portion sizes: small (A), medium (B), and large portion (C) to help estimate the amount of food consumed the previous day using the 24-h recall method. Moreover, we provided instructions and guidelines for portion estimation. Additionally, information regarding food preparation methods was collected through athletes self-reporting.

#### 2.2.3 Simple reaction time (SRT)

The processing speed was measured by evaluating the simple reaction time (SRT). Participants were given directions to rapidly press a button once a visual cue became visible on a computer display. The SRT test was administered using the Reaction, INRP free software (version 4.05) designed by Tilquin.

#### 2.2.4 Choice reaction time (CRT)

Using the same Reaction, INRP software, a colored geometric form referred to as the “target” was displayed to the participants. Subsequently, a series of variously colored geometric shapes appeared on the screen. Whenever the target appeared, participants were instructed to press a button as quickly as possible. The software measured the time elapsed between the appearance of the form and the participant's response, measured in seconds, with higher scores indicating less favorable performance.

#### 2.2.5 Vigilance test (Vt)

The Digit Cancellation Test, also known as the VT (Vigilance Test), is a valuable tool for evaluating cognitive-perceptual motor function, psychomotor speed, and sustained attention and vigilance (34). Its purpose is to assess various aspects of the prefrontal cortex's functioning, including the ability to focus attention, information processing speed, and executive functioning (35). Participants performed the test by crossing out target numbers (i.e., numbers composed of three digits) on a sheet of randomly arranged numbers. The participant's attention was evaluated based on the number of correctly detected targets, with one point awarded for each correctly identified 3-digit number within one minute.

#### 2.2.6 Mental rotation test (MRT)

The Mental Rotation Test (MRT) was conducted using OpenSesame software version 3.1 (36). In this test, participants were tasked with determining if two stimuli displayed on the screen are identical, following the principles of Shepard and Metzler (37). Each set in the test consisted of 10 items and required mental concentration, precision, and speedy manipulation. The test yielded two types of results: the time taken to process correct responses (MRT time) and the number of errors made (MRT errors).

### 2.3 Statistical analysis

The obtained data in this study were analyzed using STATISTICA software (StatSoft, France). Figures were generated using GraphPad Prism 8 (GraphPad Software, San Diego, CA, United States). For each variable, the means ± SD (standard deviation) values were determined. All data were normally distributed, confirmed by the Shapiro-Wilk test. The one-way repeated measures ANOVA (6 testing Phases) was conducted to analyze the effect of timepoints. Where appropriate, significant differences between means were tested using Tukey's HSD Post hoc test. The magnitude of the difference between age-groups was assessed using the effect size statistic (*η_p_*^2^). The criteria used to determine the effect sizes were as follows: 0.01 denoted a small effect size, 0.06 represented a moderate effect size, and 0.14 indicated a large effect size (38). Standardized effect size (Cohen's *d*) analysis was used to interpret the magnitude of differences between variables and classified them according to (39) as: trivial (*d* ≤ 0.20); small (0.20 < *d* ≤ 0.60); moderate (0.60 < *d* ≤ 1.20); large (1.20 < *d* ≤ 2.0); very large (2.0 < *d* ≤ 4.0); and extremely large (*d* > 4.0). A significant level was considered as a *p* ≤ 0.05.

## 3 Results

### 3.1 The Pittsburgh Sleep Quality Index (PSQI)

The one-way ANOVA test results indicated that Phases had a significant main effect on various sleep parameters, including sleep duration [*F* (1,22) = 47.15, *p* < 0.001, ηp2 = 0.68], sleep quality [*F* (1,22) = 64.09, *p* < 0.001, ηp2 = 0.75], sleep disturbances [*F* (1,22) = 37.11, *p* < 0.001, ηp2 = 0.62], daytime dysfunction [*F* (1,22) = 34.44, *p* < 0.001, ηp2 = 0.61], and total PSQI scores [*F* (1,22) = 79.64, *p* < 0.001, ηp2 = 0.78]. However, sleep latency [*F* (1,22) = 2.04, *p* > 0.05, ηp2 = 0.08], sleep efficiency [*F* (1,22) = 2.12, *p* > 0.05, ηp2 = 0.08] and the use of sleeping medication [*F* (1,22) = 0.21, *p* > 0.05, ηp2 = 0.009] were not significantly affected. Further Tukey test analysis revealed that sleep duration was significantly lower during all timepoints (all *p* < 0.001) when compared to R0. Moreover, compared to R0, the total PSQI scores were higher during R2, R3, R4, and R5 (all *p* < 0.001). Sleep quality, daytime dysfunction, and sleep disturbance scores increased significantly during R2, R3, R4, and R5 (all *p* < 0.001) when compared to R0 Regarding Ramadan weeks, sleep duration was lowest during R4, compared to all other periods (all *p* < 0.001) (Table 2). Indeed, the total PSQI scores (R1 (*p* < 0.001), R2 (*p* < 0.001), and R3 (*p* < 0.01)), sleep quality (all *p* < 0.001), daytime dysfunction (R1 (*p* < 0.001), R2 (*p* < 0.001), and R3 (*p* < 0.05)), and sleep disturbance scores (all *p* < 0.001) were the highest during R4 compared to all other Ramadan weeks (Table 2). Global PSQI scores were superior than 5 during R3 (6.3 ± 1.2) and R4 (7.6 ± 1.8) indicating a poor sleep quality.

**Table 2:** Mean ± SD values of PSQI questionnaire parameters, dietary intake, and body composition parameters recorded during the week before Ramadan (R0), during the 1st week of Ramadan (R1), during the 2nd week of Ramadan (R2), during the 3rd week of Ramadan (R3), during the 4th week of Ramadan (R4), and during the week following Ramadan (R5), (*n* = 23).

		R0	R1	R2	R3	R4	R5
PSQI	Sleep quality	0.5 ± 0.3^c,d,e,f^	0.7 ± 0.3^c,d,e,f^	1.5 ± 0.6^a,b,e^	1.4 ± 0.6^a,b,e^	2.2 ± 0.6^a,b,c,d,f^	1.5 ± 0.6^a,b,e^
	Sleep latency (min)	15.8 ± 2.2	16.5 ± 2.5	17 ± 3.1	17.2 ± 3.6	17.1 ± 2.9	16.4 ± 2.1
	Sleep duration (h)	7.7 ± 0.7^b,c,d,e,f^	7.2 ± 0.7^a,c,d,e,f^	6.8 ± 0.7^a,b,e^	6.6 ± 0.6^a,b,e^	6 ± 0.7^a,b,c,d,f^	6.8 ± 0.7^a,b,e^
	Sleep efficiency (%)	95.45 ± 4.17	94.25 ± 4.44	94.37 ± 3.86	94.33 ± 3.83	95.42 ± 4.06	95.05 ± 4.63
	Sleep disturbance	0.4 ± 0.3^c,d,e^	0.6 ± 0.2^c,e,f^	0.9 ± 0.2^a,b,e^	0.8 ± 0.2^a,e^	1.4 ± 0.4^a,b,c,d,f^	0.9 ± 0.4^a,e^
	Day time dysfunction	0.2 ± 0.2^c,d,e,f^	0.4 ± 0.3^c,d,e,f^	0.7 ± 0.3^a,b,e^	1 ± 0.4^a,b,c,e,f^	1.2 ± 0.5^a,b,c,d,f^	0.6 ± 0.4^a,b,d,e^
	Use of sleep medication	0 ± 0	0 ± 0	0 ± 0	0 ± 0	0 ± 0	0 ± 0
	Global score PSQI	1.9 ± 0.9^c,d,e,f^	2.4 ± 0.9^c,d,e,f^	4.2 ± 1.2^a,b,d,e^	6.3 ± 1.2^a,b,c,e,f^	7.6 ± 1.8^a,b,c,d,f^	4.8 ± 2.1^a,b,d,e^
Dietary intake	Carbohydrate (g/d)	407.4 ± 65.4^b,d,f^	414.9 ± 58.2^a^	412.5 ± 58.6	414.9 ± 58.2^a^	412.4 ± 60	414.8 ± 59.4^a^
	Protein (g/d)	66 ± 12.7	63.9 ± 16.1	69.5 ± 13	65.4 ± 15.8	67.8 ± 14.6	64 ± 16.2
	Fat (g/d)	90.3 ± 10.3	90.5 ± 10.2	88.6 ± 10.1	90.6 ± 10.2	90.2 ± 10.5	89.7 ± 10
	Energy intake (kcal/day)	2,706.7 ± 304.1	2,729.3 ± 283.3	2,725.7 ± 287.3	2,736.5 ± 288.4	2,733 ± 307.4	2,722.7 ± 285
Body composition	Body mass (kg)	62.22 ± 6.9	62.13 ± 7	62.22 ± 7.12	62 ± 7.08	62 ± 6.84	61.96 ± 7.02
	BMI (kg/m^2^)	21.7 ± 1.9	21.6 ± 1.9	21.7 ± 1.9	21.6 ± 1.9	21.6 ± 1.8	21.6 ± 1.9

^a^ (*p* < 0.05) Significant difference compared to R0.

^b^ (*p* < 0.05): Significant difference compared to R1.

^c^ (*p* < 0.05): Significant difference compared to R2.

^d^ (*p* < 0.05): Significant difference compared to R3.

^e^ (*p* < 0.05): Significant difference compared to R4.

^f^ (*p* < 0.05): Significant difference compared to R5. Global score of PSQI <5: good sleep quality, and global score of PSQI ≥ 5 up to 21: poor sleep quality.

### 3.2 Dietary intake analysis, body mass, and BMI

Our statistical analysis revealed no statistically significant variations in the players' total daily average caloric and macronutrient intake, including dietary fat (g/d), and protein (g/d) throughout the six weeks (*p* > 0.05; Table 2). However, there was a main effect of dietary carbohydrate (g/d) [*F* (1,22) = 3.78, *p* < 0.01, ηp2 = 0.14] (Table 2). Compared to R0, the dietary carbohydrate (g/d) was higher during R1, R3, and R5 (all *p* < 0.01). Moreover, there was no significant difference in players’ body mass or BMI between the six testing timepoints (all *p* > 0.05) (Table 2).

### 3.3 Simple reaction time (SRT)

**Figure 2:**
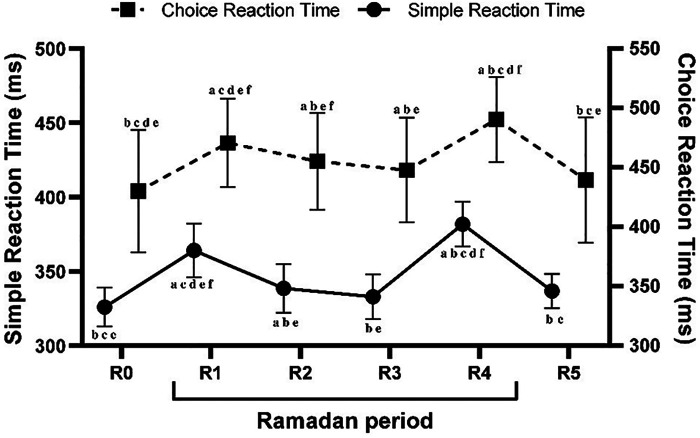
Mean ± SD values of simple reaction time (SRT) and choice reaction time (CRT) measured during the week before Ramadan (R0), during the 1st week of Ramadan (R1), during the 2nd week of Ramadan (R2), during the 3rd week of Ramadan (R3), during the 4th week of Ramadan (R4), and during the week following Ramadan (R5). ^a^(*p* < 0.05) Significant difference compared to R0. ^b^(*p* < 0.05): Significant difference compared to R1. ^c^(*p* < 0.05): Significant difference compared to R2. ^d^(*p* < 0.05): Significant difference compared to R3. ^e^(*p* < 0.05): Significant difference compared to R4. ^f^(*p* < 0.05): Significant difference compared to R5.

The one-way ANOVA test results indicated that timepoints had a significant main effect on SRT [*F* (1,22) = 66.93, *p* < 0.001, ηp2 = 0.75]. The *post hoc* test revealed that SRT was lower during R1 (*p* < 0.001), R2 (*p* < 0.05), and R4 (*p* < 0.001) compared to R0. Regarding Ramadan timepoints, in comparison with R1, SRT performance decreased during R2, R3, and R5 (all *p* < 0.001) and increased significantly during R4 (*p* < 0.001). Thus, no significant changes were reported during the R3 and R5 timepoints compared to R2 (both *p* > 0.05). Moreover, compared to R4, SRT was greater during all other timepoints (all *p* < 0.001) (Figure 2).

### 3.4 Choice reaction time (CRT)

There were significant main effects of timepoints on CRT [*F* (1,22) = 48.85, *p* < 0.001, ηp2 = 0.68]. According to the *post hoc* analysis, CRT decreased during R1 (*p* < 0.001), R2 (*p* < 0.001), R3 (*p* < 0.01), and R4 (*p* < 0.001) in comparison to R0. In comparison with R1, CRT performance decreased significantly during R2 (*p* < 0.01), R3 (*p* < 0.001), R5 (*p* < 0.001) and increased significantly during R4 (*p* < 0.001). Moreover, compared to R2, no significant changes were reported during R3 (*p* > 0.05), however, CRT was lower during R4 (*p* < 0.001) and better during R5 (*p* < 0.01). In addition, compared to R4, CRT was greater during all other timepoints (all *p* < 0.001) (Figure 2).

### 3.5 Vigilance test (Vt)

**Figure 3:**
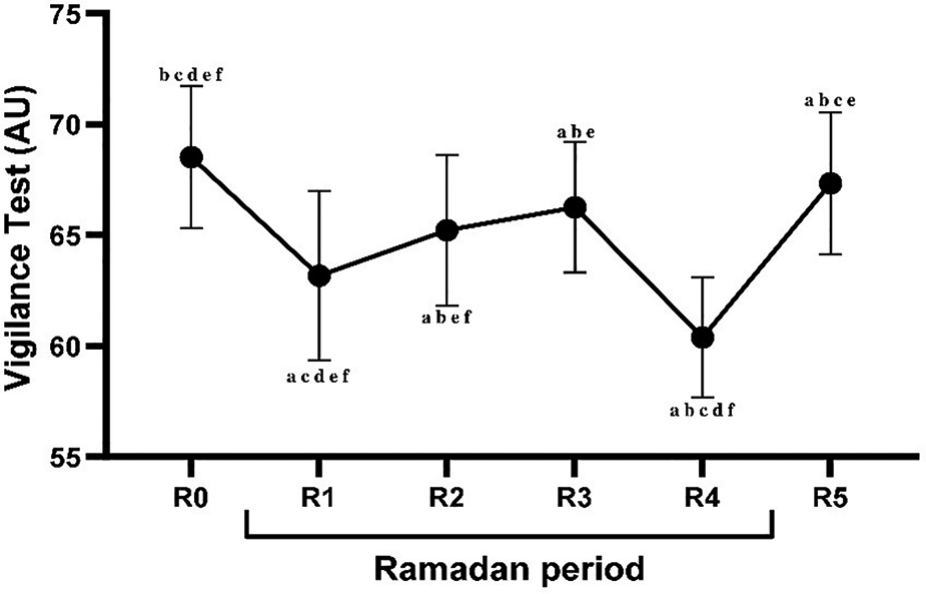
Mean ± SD values of vigilance test (VT) recorded during the week before Ramadan (R0), during the 1st week of Ramadan (R1), during the 2nd week of Ramadan (R2), during the 3rd week of Ramadan (R3), during the 4th week of Ramadan (R4), and during the week following Ramadan (R5). ^a^(*p* < 0.05) Significant difference compared to R0. ^b^(*p* < 0.05): Significant difference compared to R1. ^c^(*p* < 0.05): Significant difference compared to R2. ^d^(*p* < 0.05): Significant difference compared to R3. ^e^(*p* < 0.05): Significant difference compared to R4. ^f^(*p* < 0.05): Significant difference compared to R5.

The statistical analysis revealed significant main effects of timepoints on the VT scores [*F* (1,22) = 124.26, *p* < 0.001, ηp2 = 0.84]. The post-hoc testing identified that VT scores were significantly lower during R1 (*p* < 0.001), R2 (*p* < 0.001), R3 (*p* < 0.001), R4 (*p* < 0.001), and R5 (*p* < 0.05) compared to R0. In the context of the Ramadan timepoints, VT scores showed a significant decreased during R2 and R3) compared to R1 (all *p* < 0.001), and a significant increase during R4 (*p* < 0.001). Indeed, compared to R2, VT scores were higher during R1 (*p* < 0.001) but remained unchanged during R3 (*p* > 0.05). Furthermore, the lowest VT scores were recorded during R4 when compared to all other timepoints (all *p* < 0.001) (Figure 3).

### 3.6 Mental rotation test (MRT)

#### 3.6.1 MRT_time_

**Figure 4:**
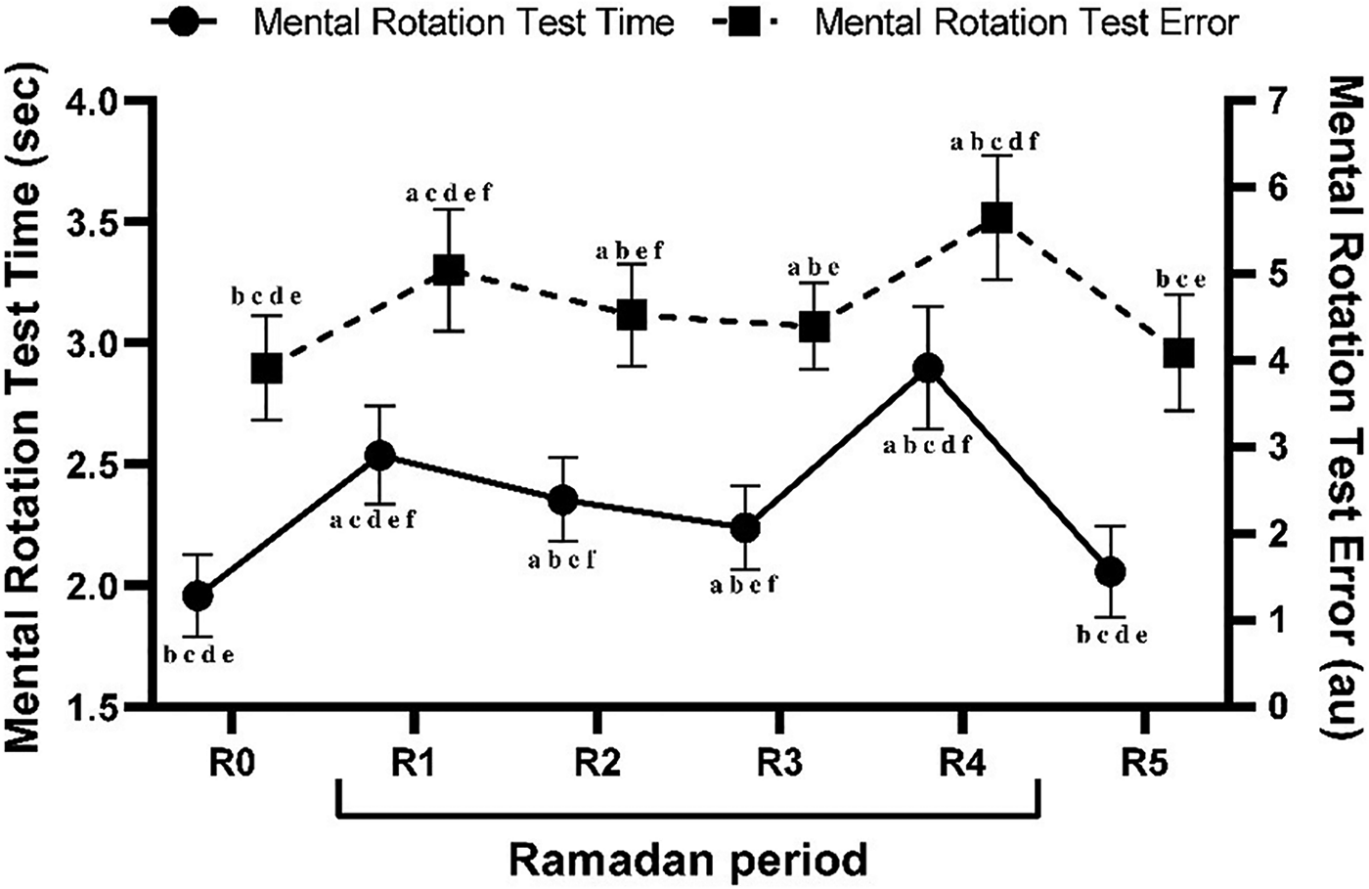
Mean ± SD values of mental rotation test time (MRT_time_) and mental rotation test errors (MRT_errors_) measured during the week before Ramadan (R0), during the 1st week of Ramadan (R1), during the 2nd week of Ramadan (R2), during the 3rd week of Ramadan (R3), during the 4th week of Ramadan (R4), and during the week following Ramadan (R5). ^a^(*p* < 0.05) Significant difference compared to R0. ^b^(*p* < 0.05): Significant difference compared to R1. ^c^(*p* < 0.05): Significant difference compared to R2. ^d^(*p* < 0.05): Significant difference compared to R3. ^e^(*p* < 0.05): Significant difference compared to R4. ^f^(*p* < 0.05): Significant difference compared to R5.

The results from the one-way ANOVA demonstrated a significant main effect of timepoints on MRT_time_ [*F* (1,22) = 117.78, *p* < 0.001, ηp2 = 0.84]. Post hoc analysis revealed that MRT_time_ was significantly longer during R1, R2, R3, and R4 compared to R0 (all *p* < 0.001). Regarding Ramadan timepoints, in comparison with R1, MRT_time_ performance decreased during R2 (*p* < 0.01), R3 (*p* < 0.001), and R5 (*p* < 0.001) and increased significantly during R4 (*p* < 0.001). Therefore, compared to R2, MRT_time_ was better during R1 (*p* < 0.01) but it remained unchanged during (R3 *p* > 0.05). Additionally, compared to R4, MRT_time_ was greater during R1, R2, R3, and R5 (all *p* < 0.001) (Figure 4).

#### 3.6.2 MRT_errors_

Statistical analysis revealed significant effects of timepoints on MRT_errors_ [*F* (1,22) = 41.50, *p* < 0.001, ηp2 = 0.65]. *Post hoc* test identified that MRT_errors_ scores were higher during R1 (*p* < 0.001), R2 (*p* < 0.001), R3 (*p* < 0.05), and R4 (*p* < 0.001) compared to R0. Regarding Ramadan timepoints, compared with R1, MRT_errors_ decreased during R2 (*p* < 0.01), and R3 (*p* < 0.001), and increased significantly during R4 (*p* < 0.001). Additionally, when compared to R2, MRT_errors_ scores were higher during R1 (*p* < 0.01) and R5 (*p* < 0.05), but remained unchanged during R3 (*p* > 0.05). Moreover, MRT_errors_ were lower during all other timepoints compared to R4 (all *p* < 0.001) (Figure 4).

## 4 Discussion

Every year, healthy adult Muslims refrain from eating and drinking from dawn to sunset for one whole month during IF. This practice, which aligns with the ninth month of the Islamic lunar calendar, represents the fourth pillar of Islam and is a unique variant of intermittent fasting. While IF has been increasingly recognized for its health benefits, recent findings indicate the positive effects of RIF model on several physiological markers. Recent systematic reviews and meta-analyses revealed that RIF induces significant positive changes in body weight and composition (40, 41), liver function (42), cardiometabolic markers (43), glucometabolic markers (44), metabolic syndrome components (45), and inflammatory and oxidative stress markers (46) in the healthy population. Furthermore, the concurrence of sporting events and competitions with the Ramadan fasting period necessitates a comprehensive understanding of the physiological effects of fasting on athletes. This includes an examination of various bodily functions such as cognitive performance, metabolic and cardiovascular responses, physical performance, and alterations in sleep patterns and alertness (47, 48).

Therefore, this study aimed to investigate the effects of RIF on neuromuscular and cognitive performance in adolescent female team ball players. The primary outcomes of the study indicated that: (i) cognitive performance was adversely impacted by Ramadan fasting, both during and after the fasting month; (ii) variations in cognitive performance were associated with different periods of Ramadan; (iii) Ramadan fasting did not significantly alter daily energy intake or body composition; and (iiii) sleep quality was notably compromised by Ramadan fasting, particularly during the final week of the fasting period.The present study results revealed that Ramadan fasting impairs cognitive performance. Our findings are in line with previous studies that indicated that RIF has a detrimental impact on processing speed (20), attention (19, 49), and simple reaction time (15). Indeed, several investigations found no correlation between RIF and short-term memory performance (12), motor reaction time (14), choice reaction time (15, 16), simple reaction time (16, 17), or attention (18). Yet, to the best of our knowledge, only three studies examined these effects in female athletes, two of which focused on young handball players (4, 18) and the other on young sprinters (12). According to the findings of these latter studies, the RIF was not associated with an adverse effect on alertness or vigilance, however, sleep quality was not assessed during these studies. Aside from the variations in motivation and different chronotypes (sleep and activity preferences, which is neither chronotype (intermediate) in our study (not an extreme morning nor evening chronotype), fasting duration, which is influenced by the local environmental conditions (such as temperature, humidity, and season) (4), could elucidate the negative effect of RIF on cognitive functions and the absence of any discernible adverse effects in some studies. Moreover, discrepancies in participant characteristics, including sex, age, fitness levels, and individual physical activity, may contribute to the inconsistency in findings observed across these diverse studies. Our study's findings elucidate the multifaceted impact of RIF on cognitive and psychomotor performance in young athletes, particularly females. The impairment in cognitive performance observed in the afternoon is multifactorial. Firstly, the decline in performance noted in the afternoon can be attributed to the fasting duration, which lasted approximately 15.5 h in our study. This prolonged fasting can result in reduced glycogen stores, decreased blood glucose levels more than 6 h after the last meal (50), and an overall decrease in energy availability, contributing to fatigue and diminished performance (51). Additionally, the impairment in performance we noticed during RIF may also be attributable to diminished blood glucose levels, as glucose is a crucial resource for the central nervous system and other studies have indicated an increased metabolism of glucose in particular brain regions during cognitive tasks (17). Since the brain cannot generate its own glucose and depends on a continuous supply from the periphery (which is lower in fasting individuals during the late afternoon), this decrease in blood glucose levels in the afternoon could be the underlying cause of the deterioration in cognitive performance (4).

The decline in cognitive skills related to RIF could be partially explained by the lack of or inadequate predawn meal (Suhoor or Sahour) timing, quantity, and/or quality among athletes. Recent findings in female athletes indicate the association between nutrition timing during RIF and the variation in cognitive (2) and physical (52) performance throughout the day, particularly at midday and in the afternoon. A late last pre-dawn meal was advantageous to preserve better cognitive and physical performance in the morning and prevent any decrease during the midday or afternoon fast. Interestingly, this decline in cognitive performance found in the current study could neither be attributed to the decrease in calorie consumption nor to the changes in the body compositions among athletes. Our results showed that RIF was not associated related to variations in total daily caloric intake at different timepoints, which is consistent with previous studies among female athletes (5, 18, 52, 53). Similarly, Abdelrahim et al. (54) indicated that the different physiological and health effects associated with RIF may be explained by changes in meal timing rather than by changes in quantitative dietary intake. Nevertheless, Jahrami et al. (41) suggested that RIF might be linked to a significant, small decrease in body weight in young, healthy, non-athletic individuals. In the athletic population, while Trabelsi et al. (55) revealed that RIF could lead to a significant decrease in body mass among adult athletes, Aloui et al. (56) revealed no relationship between RIF and any potential effects on body composition.

Additionally, RIF may influence the daily performance pattern through a variety of mechanisms, including decreasing performance amplitude rhythm, causing a shift in phase advance or delay in the rhythm (4, 57), impacting athletes' motivation and mood, as well as changes in the levels of a number of hormones, including leptin, adiponectin, ghrelin, cortisol, and melatonin (58). Similarly, all of these changes may be quite significant for diminishing psychomotor and cognitive functions during this month. Furthermore, it has recently been suggested that the decline in performance may not be caused by the fasting effect but rather by the fatigue imposed by repetitive partial sleep deprivation observed during the end of RIF (4, 11). These latter findings align with our results and a recent meta-analysis (6) that indicated a significant alteration in sleep duration and a decline in sleep quality. The decrease in sleep duration can be attributed not only to shifts in meal times and the consumption of large quantities of food late at night, but also to lifestyle changes that disrupt the body's natural biological clock during this month. The tiredness resulting from this partial sleep deprivation may account for the decline in afternoon cognitive performance during Ramadan. In this context, in line with our current findings, it has been demonstrated that sleep deprivation can result in a decline in attentional abilities and slower reaction times, particularly noticeable in the late afternoon (59, 60). Knowing that it's important to note that getting less than the recommended eight hours of sleep can lead to cognitive performance deficits, this phenomenon could be a plausible explanation for the reduced alertness we observed (61). In our study, the average estimated sleep duration, especially during the last week, was considerably below the recommended average, measuring approximately 6 h with a sleep duration of approximately 100 min. However, it's worth noting that conflicting results exist regarding the impact of RIF on cognitive functions. Indeed, in most studies that did not find negative effects on sleep quality during Ramadan fasting, there were no observed decrements in cognitive performance either (15–17, 62). These findings could offer some insight, albeit partially, into the reduced cognitive performance observed in the current study in relation to sleep patterns. Hence, it is suggested that the adverse impact of RIF in physical performance is predominantly attributed to the placebo (or nocebo) effects associated with the act of observing the fast itself (62). We hypothesize that the performance impairment during RIF may be partially explained by the Nocebo effect on cognitive processes. Nocebo effects results from negative beliefs regarding the results of a treatment or action, such as fasting during Ramadan in this context (63). The nocebo response may have been enhanced in our study's athletes who had previously fasted throughout RIF because of their prior fasting experiences. Before Ramadan, 86.9% of participants indicated it might have a detrimental effect on cognitive function. Consistent with prior studies (4, 11) and a recent meta-analysis (56), our findings align. There's no notable shift in daily energy intake or body composition during Ramadan. This suggests that rather than changes in calorie consumption, the performance decline may be related to less sleep during this month.

Regarding the fluctuations in cognitive performance during various phases of Ramadan, our study revealed a gradual recovery in these cognitive abilities, starting from R2 to R3. This observation suggests that athletes' cognitive functions exhibit an adaptive response over time. This outcome aligns with previous studies (14, 21), which demonstrated that total reaction time and recognition reaction time were affected primarily at the outset of RIF, with initial adverse effects on reaction times and stability toward the end of Ramadan fasting. This hints at the potential for adaptation to intermittent fasting. However, the adaptation that was evident during R2 and R3 seemed to diminish and even disappear during the final week. We suggest that these changes in adaptation response could be attributed to a combination of factors, including cumulative fatigue, inadequate sleep, nutrition deficiencies, dehydration, disrupted circadian rhythms, and psychological stress related to the final week of Ramadan. As Ramadan progresses, these factors, when compounded, can significantly impact an athlete's ability to maintain optimal cognitive performance.

## 5 Study limitations and implications for future research

Despite its findings, the current study highlights certain limitations that should be taken into account. It is worth noting that the current study did not involve a control group. The aforementioned limitation is an important characteristic of many investigations on RIF because the majority of these investigations were conducted in Muslim countries. In these contexts, recruiting non-fasting individuals for studies is regrettably impractical for ethical reasons. As a result, a common approach involves comparing RIF measurements to the values before Ramadan, which are often used as reference points, as commonly observed in the literature. Furthermore, all assessments were conducted under resting conditions rather than during physical exertion. In this aspect, it would be valuable to explore how RIF affects the cognitive function measurements of athletes while they are actively exercising. Additionally, blood-related data were not collected in the current study, preventing an investigation into the potential influence of metabolic and hormonal factors on cognitive performance. Furthermore, sleep parameters were exclusively gathered through the subjective use of the PSQI, not with objective tools. The timing, quantity, and quality of the consumed last pre-dawn meal and the water/fluid intake were not controlled in the current study. Lastly, the research focused exclusively on the female sex, where hormonal fluctuations and various other factors could play a significant role. Consequently, it would be beneficial to compare these findings with data obtained from male participants to understand any sex-specific differences or similarities in the effects observed. The observational nature of the current work with the lack of controlled group and controlled dietary and lifestyle behaviors make it difficult to infer causality and to ascribe the changes to RIF alone.

## 6 Conclusion

The findings of our study highlight the significant impact of RIF on the cognitive and psychomotor performance of young female athletes, without corresponding changes in dietary intake or body composition. This suggests that the detrimental effects observed are predominantly due to factors like cumulative fatigue and reduced sleep quality, particularly in the final week of fasting. These insights provide a valuable practical implication for coaches, trainers, and athletes: prioritizing sleep quality and managing cumulative fatigue during Ramadan is essential.

To support athletes observing Ramadan, it is advisable to develop structured sleep hygiene programs and fatigue management strategies. These could include improving sleep quality, adjusting training schedules to enhance rest periods, and teaching athletes about the importance of consistent sleep patterns. Incorporating mindfulness and relaxation techniques could also help in reducing psychological stress, thereby enhancing overall well-being and performance. Considering the potential for adaptation to fasting, a phased training approach that aligns with the stages of Ramadan could be advantageous. Such an approach would involve initially maintaining base fitness levels and gradually increasing intensity to prevent overburdening athletes as they adapt to fasting conditions. Future studies are warranted to explore these areas in greater depth in a larger population with both sexes being included.

## Data Availability

The original contributions presented in the study are included in the article/Supplementary Material, further inquiries can be directed to the corresponding author.
